# Efficacy and safety of immune checkpoint inhibitors rechallenge in advanced solid tumors: a systematic review and meta-analysis

**DOI:** 10.3389/fonc.2024.1475502

**Published:** 2024-12-12

**Authors:** Jiahui Cao, Xinyao Ding, Jianan Ji, Lin Zhang, Chengyan Luo

**Affiliations:** Department of Gynecology, First Affiliated Hospital with Nanjing Medical University, Nanjing, China

**Keywords:** immune checkpoint inhibitors, rechallenge, solid tumors, immunotherapy, efficacy, safety

## Abstract

**Introduction:**

The efficacy and safety of re-administration of immune checkpoint inhibitors (ICIs) in advanced solid tumors lacks consensus and is of great concern to clinicians. This study aimed to investigate the efficacy and adverse effects of ICIs rechallenges in advanced solid tumors.

**Methods:**

We systematically searched the databases of PubMed, Embase, the Cochrane Library, and the China National Knowledge Infrastructure (CNKI), and screened the relevant literature according to the inclusion and exclusion criteria. Meanwhile, we conducted a meta-analysis of objective response rates (ORR), disease control rates (DCR), and immune-related adverse events (irAEs) for reuse of ICIs using Freeman-Tukey double arcsine transformation method.

**Results:**

Sixty eligible studies were included in the meta-analysis, and the results revealed that those who discontinued ICIs therapy and reused ICIs had an ORR of 21.6% [95% confidence interval (CI): 17.6, 25.7] and a DCR of 55.8% (95% CI: 50.0, 61.5). The overall incidence for grade ≥ 3 irAEs was 16.7% (95% CI: 11.8, 22.2). In the subgroup analysis, patients with renal cell carcinoma presented superior efficacy with an ORR of 30.9%, which was higher than that of melanoma (24.3%) and non-small cell lung cancer (NSCLC) (10.1%). Patients who have been treated with single-agent ICIs, re-treatment with a combination of ICIs directing different targets presents better outcomes, with ORR of 22.5% and DCR of 38%, respectively, compared with those patients who continue to use a single agent.

**Conclusion:**

Patients with advanced solid tumors who have relapsed or progressed after prior treatment with ICIs may benefit from ICIs rechallenge, with a comparable incidence of grade ≥ 3 irAEs to those previously treated with ICIs.

**Systematic review registration:**

https://www.crd.york.ac.uk/PROSPERO/, identifier CRD42023407409.

## 1 Introduction

Advanced solid tumors, such as advanced lung cancer, melanoma and cervical cancer, have a poor prognosis, with 5-year survival rates of 6%, 10% and 17%, respectively (1–3). The use of immune checkpoint inhibitors (ICIs) represents a major breakthrough in the treatment of advanced tumors, significantly improving the prognosis in a wide range of advanced solid tumors. After treatment with ICIs alone, the 5-year overall survival (OS) rate for advanced lung cancer can be as high as 42.9% (4), and the objective response rate (ORR) for recurrent or metastatic melanoma and cervical cancer can reach 52% and 26.3%, respectively (5, 6). ICIs offer a promising future for patients with solid tumors, but are frequently discontinued in clinical practice due to tumor progression and serious immune-related adverse events (irAEs), which occur at rates of 40% and 40%, respectively (7–9). After interruption of this treatment, the available options (including radiation and chemotherapy) are limited and ineffective (10, 11). For patients with advanced solid tumors, the opportunity to repeat a potentially effective ICIs, known as “ICIs rechallenge”, with robust safety is a major concern for clinicians. However, to date, there are fewer relevant prospective clinical studies, and no consensus has been reached.

Various regimens are available for ICIs rechallenge, such as ICIs on different targets, or multi-target ICIs, or combination of different target ICIs, or combination of ICIs with anti-angiogenic drugs, or combination of ICIs with chemotherapy, or combination of ICIs with radiotherapy. The efficacy of these regimens and treatment-related adverse effects (TRAE) have been inconsistently reported. Ribas et al. included 359 patients with advanced melanoma with disease progression after the use of ipilimumab and compared the efficacy of chemotherapy, anti-programmed cell death-1 (PD-1) inhibitor at 2 mg/kg, and anti-PD-1 antibody at 10 mg/kg, and found that reapplication of ICIs showed superior tumor control compared to chemotherapy, with ORRs of 23.4% and 4.5%, respectively, and the incidence of TRAEs of grade 2 or higher was lower (12.6% vs. 26.3%) (12). This study demonstrates that ICIs rechallenge can still benefit and be safe for patients with advanced melanoma who have previously used ICIs. In contrast, Bowyer et al. analyzed 40 patients with advanced malignant melanoma who were rechallenged with an ICIs acting on a different target after progression with an ICIs and found an ORR of only 10% (13).

For patients with advanced solid tumors previously treated with ICIs, ICIs rechallenge is one of the options after disease progression, but there is a lack of prospective randomized controlled trials (RCTs) with large samples to confirm the efficacy and safety. Therefore, we used a single-arm meta-analysis to investigate the efficacy and adverse effects of ICIs retesting in patients with advanced solid tumors to provide a basis for clinical decision-making.

## 2 Materials and methods

### 2.1 Search strategy

We searched the relevant literature in the following databases: PubMed, EMBASE, the Cochrane Library and Chinese National Knowledge Infrastructure (CNKI). The last search date was 1 August 2023. The following terms were used in combination with Boolean operators (AND, OR, NOT): subject and free words for “neoplasms” and “Immune Checkpoint Inhibitors” AND (rechallenge OR retreatment OR restart OR reuse). A sample of the PubMed search technique was provided in **Supplementary Material Table S1**. The protocol was registered in the International Prospective Register of Systematic Reviews (PROSPERO) with registration number CRD42023407409, https://www.crd.york.ac.uk/PROSPERO/display_record.php?RecordID=407409.

### 2.2 Study screening

After the systematic search, all literature was manually screened to identify eligible studies. Two reviewers (JC and XD) independently reviewed the titles and abstracts of the articles to identify eligible full-texts. After screening, the literature that met the inclusion criteria was enrolled. In the case of disagreement, they discussed it with each other or relied on a third researcher (JJ) to make a judgement. The inclusion criteria were as follows: (1). All studies other than reviews were eligible for inclusion, including prospective, retrospective, cross-sectional and cohort studies (2). Studies of ICIs rechallenge after discontinuation of ICIs therapy due to progressive disease (PD), completion of treatment, or irAEs were included (3). Studies that reported tumor control outcomes during treatment, including ORR and disease control rate (DCR), or TRAEs, were included (4). Those studies with sample sizes greater than 5 were included. The exclusion criteria were as follows: non-human study, no results that we needed, and full text not available or duplicated.

### 2.3 Literature quality assessment and data extraction

We used the MINORS index to evaluate the quality of the included literatures (14). MINORS involves eight items for non-comparative studies and 12 items for comparative studies and the maximum item score is 2. The general section of the scale contains eight items, including a clearly stated aim, inclusion of consecutive patients, prospective collection of data, endpoints appropriate to the aim of the study, unbiased assessment of the study endpoint, follow-up period appropriate to the aim of the study, loss of follow-up less than 5% and prospective calculation of the study size. Four additional items were added to the comparative study, including an adequate control group, contemporary groups, baseline equivalence of groups, and appropriate statistical analyses. Two reviewers (JC and LZ) assessed the quality of the literature independently, and the disagreements were resolved by discussion with a third researcher (JJ). A total score of more than eight was considered to be of satisfactory quality (14). All necessary data for the study were extracted and collated independently by two researchers. Data on authors, year of publication, study design (including type of design and sample size), tumor outcomes (including ORR and DCR), and TRAEs incidence and severity were extracted for meta-analysis from the eligible articles.

### 2.4 Statistical analysis

All the data in this meta-analysis were analyzed with R 4.3.0. software. To correct for extreme ratios (e.g., less than 0.2 and greater than 0.8) and small sample sizes, Freeman-Tukey double arcsine transformation was employed (15). Heterogeneity was measured using the chi-squared test and the I^2^ statistic. p < 0.1 indicated a statistically significant difference. If there was significant heterogeneity (p-value less than 0.1 and I^2^ greater than 50%), a random-effects model was used, otherwise a fixed effects model was adopted (15, 16). Publication bias was assessed utilizing funnel plots (17).

## 3 Results

### 3.1 Study screening

**Figure 1:**
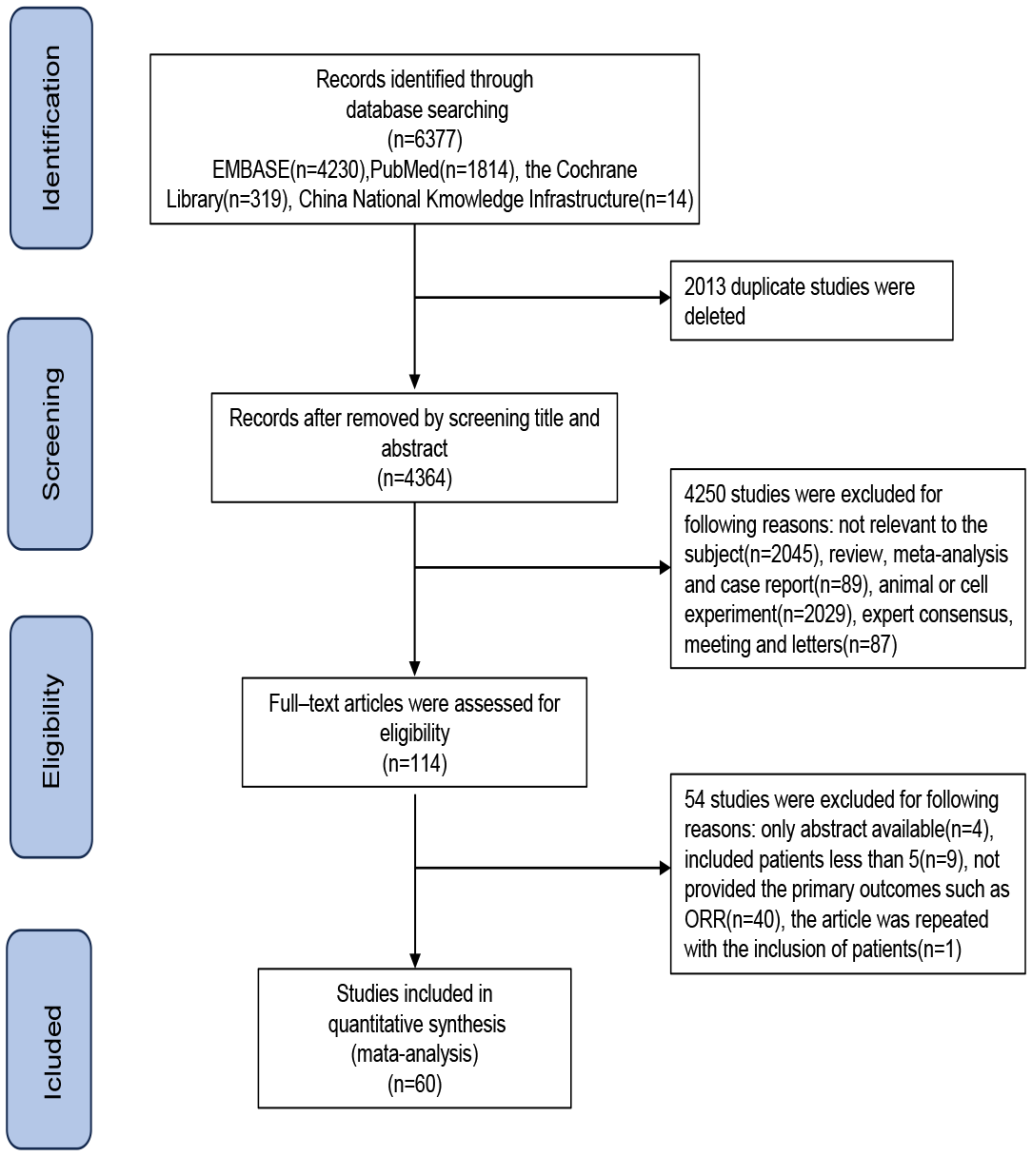
Study selection flowchart.

Preliminary search results show a total of 6,377 published studies across the four databases. After excluding duplicate studies, studies with small sample sizes, and studies lacking full text and treatment outcomes, 60 studies that met the inclusion criteria were ultimately included in the meta-analysis. The literature screening flowchart is shown in **Figure 1**. Details of each included study are summarized in **Supplementary Material Table S2**.

### 3.2 Tumor response in ICIs rechallenge

**Figure 2:**
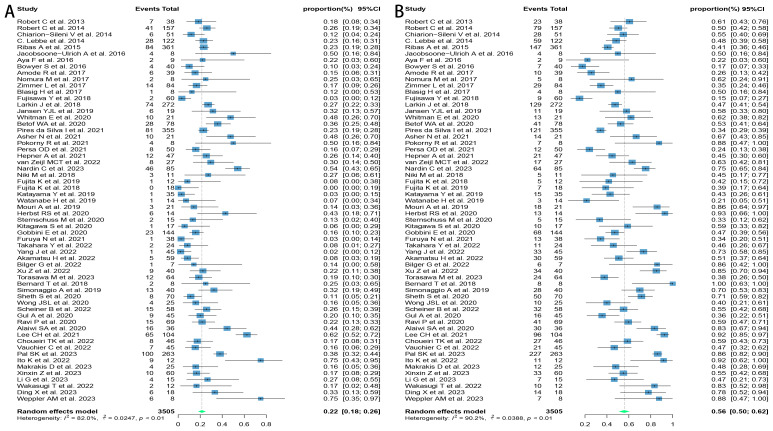
Forest plot of response after rechallenge with ICIs in patients with advanced solid tumors. **(A)** The ORR for reuse of ICIs; **(B)** The DCR for reuse of ICIs. ICIs, immune checkpoint inhibitors; ORR, objective response rate; DCR, disease control rate.

Sixty studies covering melanoma, lung cancer, renal cell carcinoma, hepatocellular carcinoma, uroepithelial carcinoma, gastric cancer, cervical cancer, and other solid tumors were included in this review, which were tested to be significantly heterogeneous (I^2^ = 83.3%, p < 0.01), and therefore meta-analyzed using a random-effects model. The results found that the ORR and DCR for reuse of ICIs in patients with advanced solid tumors were 21.6% [95% confidence interval (CI): 17.6, 25.7] and 55.8% (95% CI: 50.0, 61.5), respectively (**Figure 2**).

**Figure 3:**
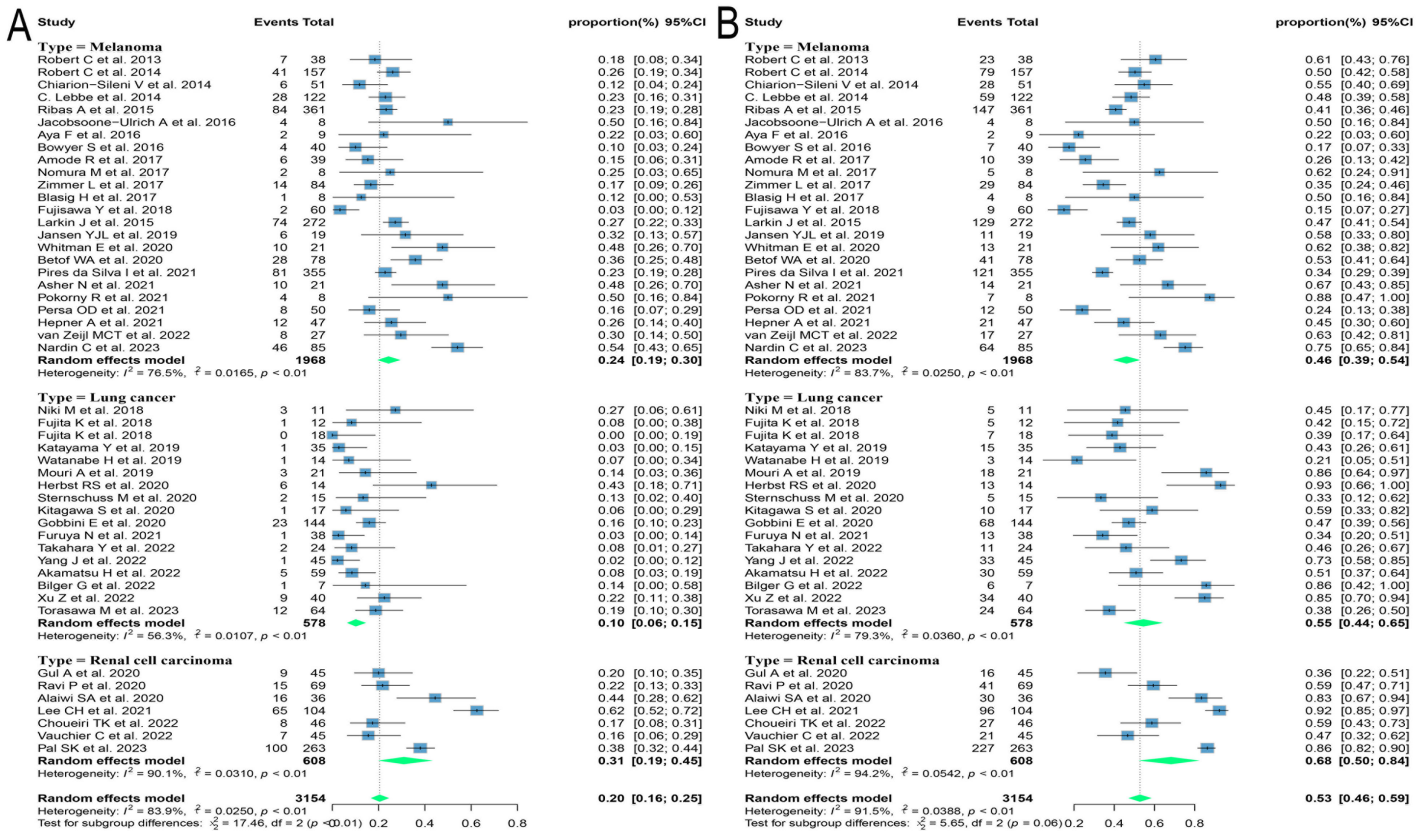
Forest plot of subgroup analysis of efficacy after rechallenge with ICIs in patients with advanced solid tumors based on different histological types. **(A)** The ORR for reuse of ICIs; **(B)** The DCR for reuse of ICIs. ICIs, immune checkpoint inhibitors; ORR, objective response rate; DCR, disease control rate.

Due to the variety of histological types involved in this study, further subgroup analyses were performed on those with three or more relevant studies of the corresponding histological type. The results found that patients with melanoma and renal cell carcinoma had better outcomes, with ORRs of 24.3% (95% CI: 18.9, 30.1) and 30.9% (95% CI: 18.7, 44.6) and DCRs of 46.1% (95% CI: 38.7, 53.6) and 68.3% (95% CI: 50.5, 83.8), respectively. Patients with lung cancer had poorer outcomes, with ORR and DCR of 10.1% (95% CI: 5.9, 15.1) and 54.5% (95% CI: 43.7, 65.1), respectively (**Figure 3**).

**Figure 4:**
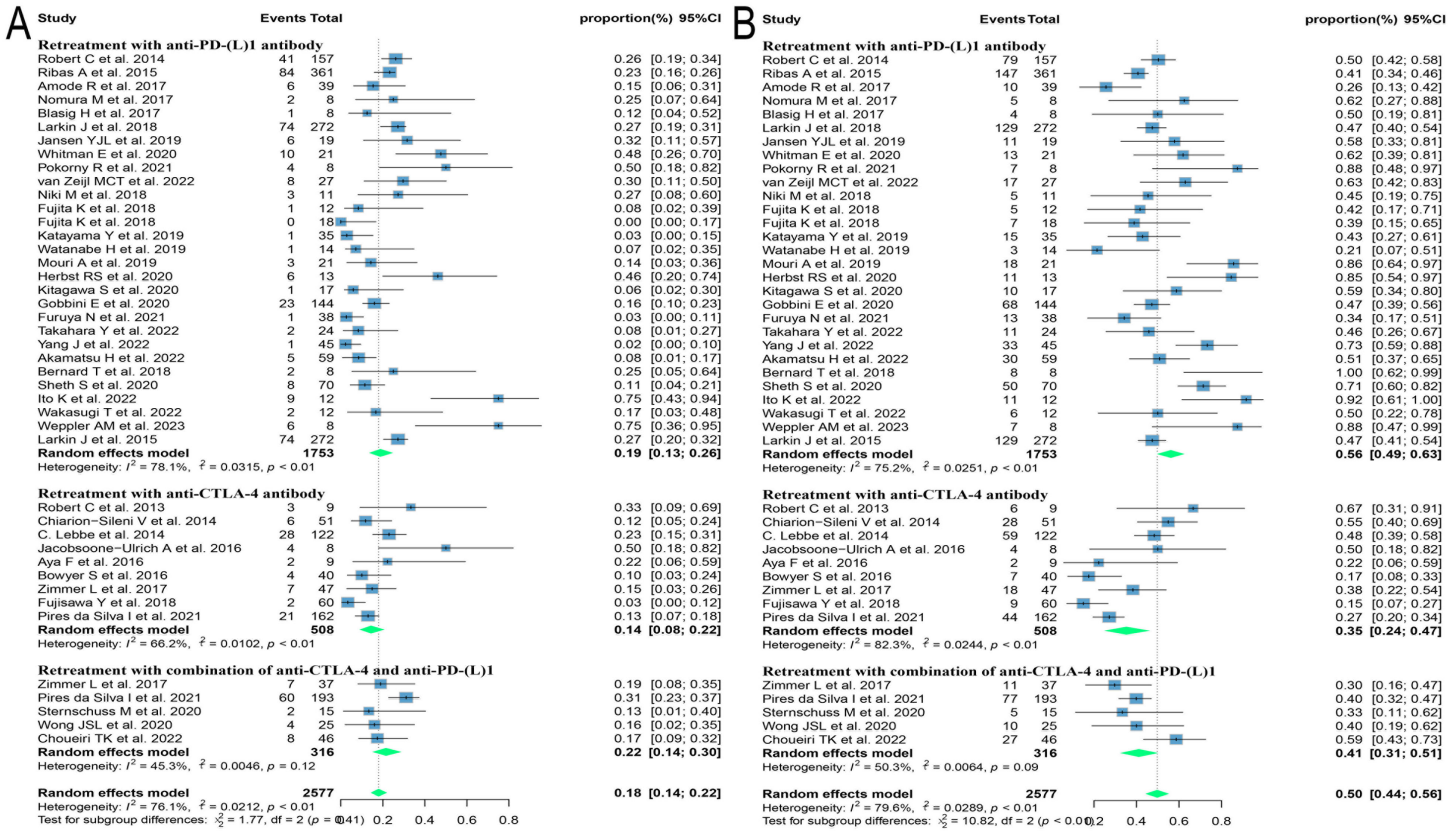
Forest plot of subgroup analysis of efficacy after rechallenge with ICIs in patients with advanced solid tumors based on different regimens at rechallenge, including anti-CTLA-4 antibody, anti-PD-(L)1 antibody, and combination of anti-CTLA-4 and anti-PD-(L)1 antibodies. **(A)** The ORR for reuse of different ICIs; **(B)** The DCR for reuse of different ICIs. ICIs, immune checkpoint inhibitors; ORR, objective response rate; DCR, disease control rate.

**Figure 5:**
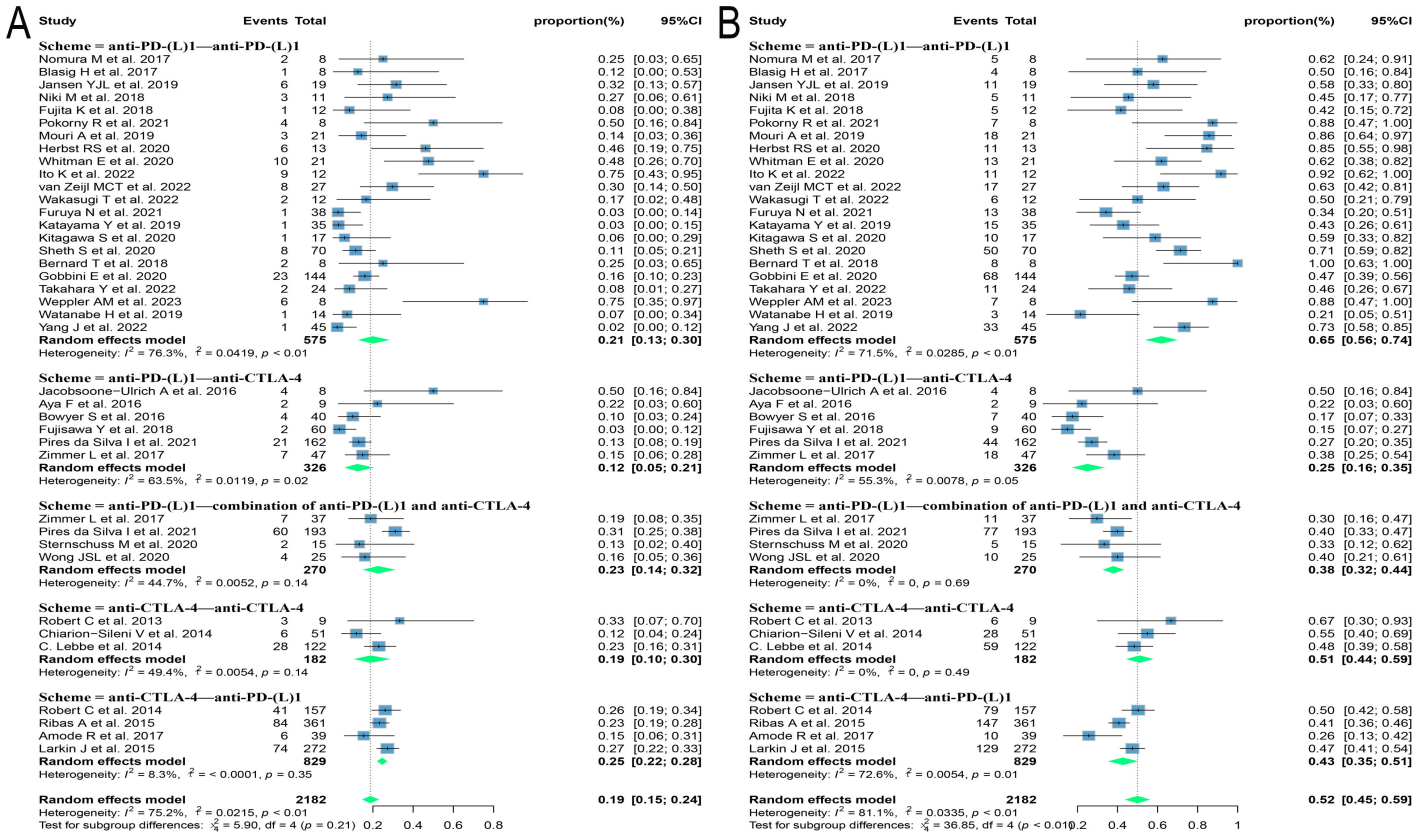
Forest plot of subgroup analysis of efficacy after rechallenge with ICIs in patients with advanced solid tumors based on different regimens at the time of initial and rechallenge treatment. **(A)** The ORR for reuse of ICIs; **(B)** The DCR for reuse of ICIs. ICIs, immune checkpoint inhibitors; ORR, objective response rate; DCR, disease control rate.

Different regimens used at re-challenge can also potentially impact efficacy. Of the studies we included 29 used anti-programmed cell death protein-(ligand)1 (PD-(L)1) antibodies, 9 used anti-cytotoxic T-lymphocyte-associated antigen-4 (anti-CTLA-4) antibodies, and 5 combined anti-CTLA-4 with anti-PD-(L)1 antibodies at re-challenge. After meta-analysis, the ORRs of the above three regimens were 18.8% (95% CI: 12.8, 25.5), 14.4% (95% CI: 8.3, 21.5), and 21.5% (95% CI: 14.3, 29.6), and the DCRs were 56.2% (95% CI: 48.9, 63.3), 35.2% (95% CI: 23.7, 47.5) and 41.1% (95% CI: 31.4, 51.2), respectively (**Figure 4**). Consequently, it was shown that the combination of antibodies with different targets could improve ORR. To explore the impact of different initial treatment regimens for ICIs on efficacy during ICIs rechallenge, Subgroup analyses based on different initial treatment regimens and rechallenge regimens were also conducted in this study. For previous ICIs treatment, anti-PD(L)-1 or anti-CTLA-4 antibodies were used in 33 and 7 studies, respectively, and 20 studies did not delineate a specific regimen. Of these 33 studies, 23 analyzed the efficacy of continuing to use a single-agent anti-PD-(L)1 antibody at the time of retreatment with ICIs, and found that the ORR in this population was 20.7% (95% CI: 12.6, 30.0) and the DCR was 65.0% (95% CI: 55.5, 73.9). Six studies switched to anti-CTLA-4 antibodies at the time of ICIs retreatment, with ORR and DCR of 12.3% (95% CI: 5.2, 21.3) and 25.0% (95% CI: 16.4, 34.5), respectively. Four studies combining anti-PD-(L)1 and anti-CTLA-4 antibodies for ICIs retreatment showed an ORR of 22.5% (95% CI: 14.0, 32.2) and a DCR of 38.0% (95% CI: 32.0, 43.9), which were higher than those of anti-CTLA-4 antibodies alone. Of the seven studies that used single-agent anti-CTLA-4 antibodies for prior treatment, three studies continued to use anti-CTLA-4 antibodies alone for re-ICIs treatment, with ORR and DCR of 18.8% (95% CI: 9.7, 29.8) and 51.1% (95% CI: 43.6, 58.6), respectively, whereas analysis of the other four studies that switched to anti-PD-(L)1 antibody revealed an ORR of 24.6% (95% CI: 21.7, 27.6) and a DCR of 42.7% (95% CI: 34.5, 51.1) (**Figure 5**). These results suggest that in patients who have been treated with single-agent ICIs, re-treatment with a combination of ICIs directing different targets presents better outcomes than in patients who continue to use a single agent. The study also showed that when resistance or progression occurred after prior treatment with either anti-PD-(L)1 or anti-CTLA-4 antibodies and ICIs was reintroduced, patients who chose anti-PD-(L)1 antibodies had a higher ORR than those who used anti-CTLA-4 antibodies.

### 3.3 Immune-related adverse effects in ICIs rechallenge

**Figure 6:**
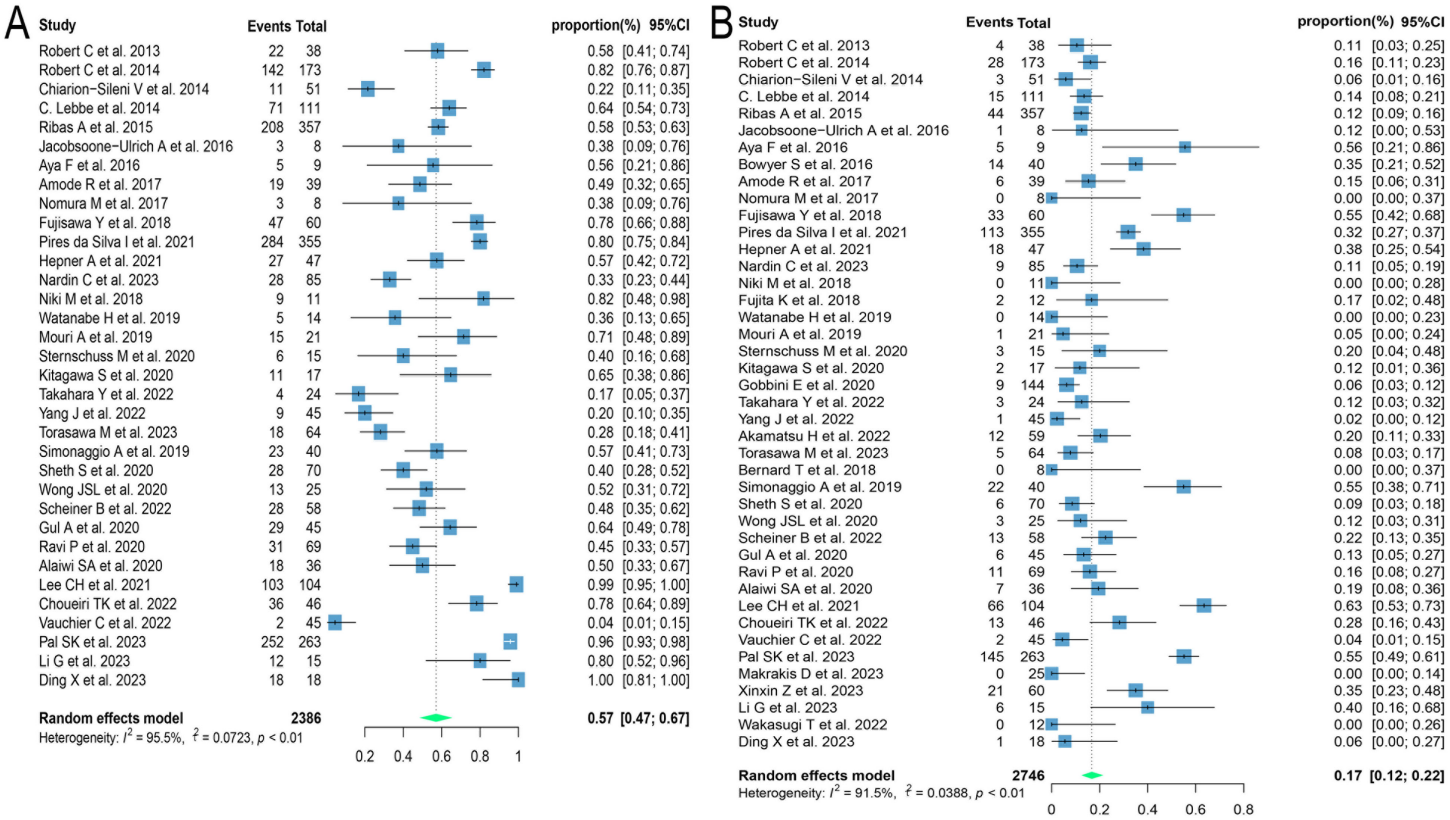
Forest plot of overall incidence of irAEs in patients with advanced solid tumors retreated with ICIs. **(A)** The incidence of any grade irAEs; **(B)** The incidence for grade ≥ 3 irAEs. irAEs, immune-related adverse events.

**Figure 7:**
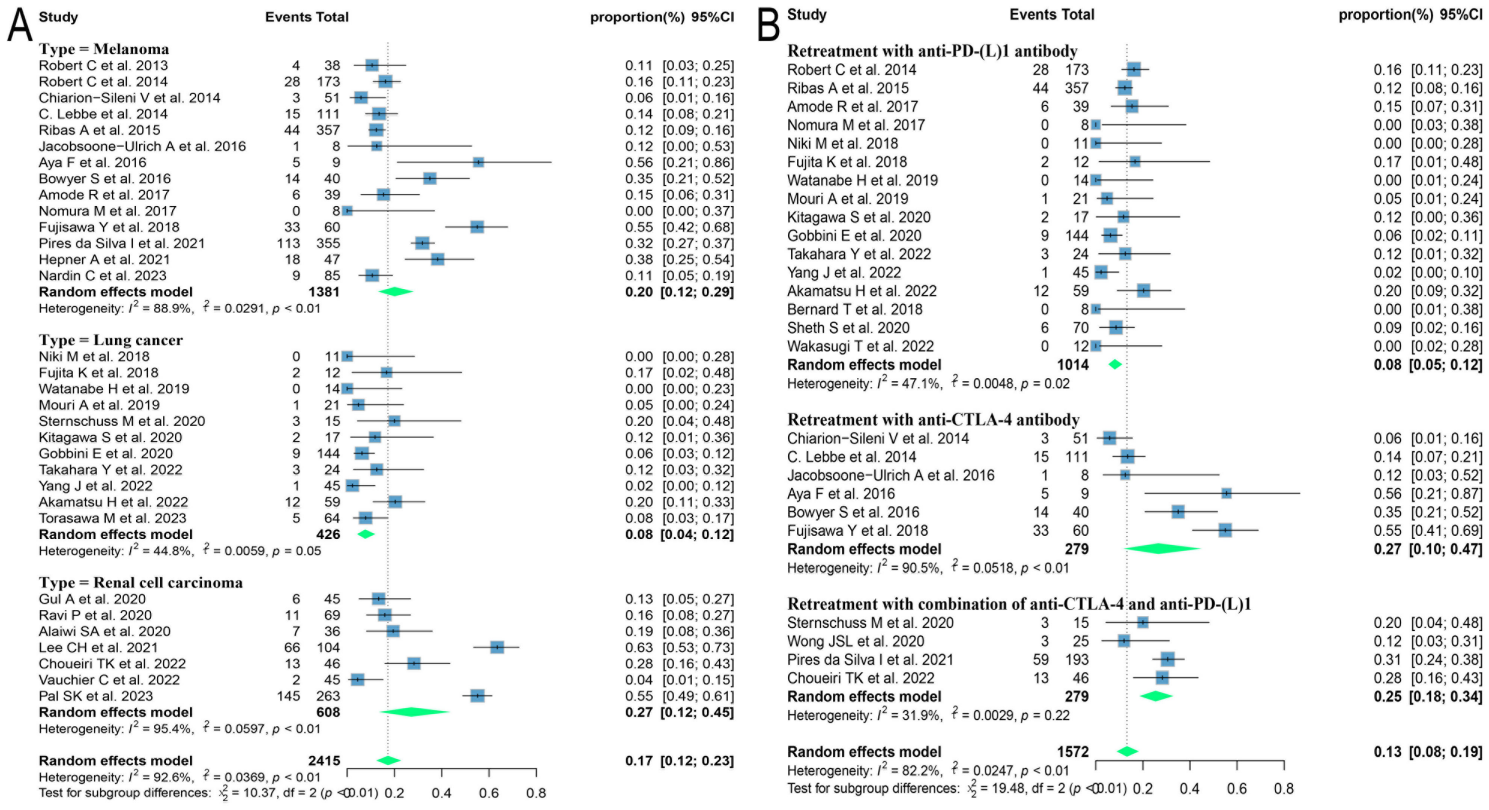
Forest plot of subgroup analysis of the incidence for grade ≥ 3 irAEs in patients with advanced solid tumors retreated with ICIs. **(A)** Subgroup analysis for different tumor histological types; **(B)** Subgroup analysis for different regimens at rechallenge. irAEs, immune-related adverse events.

Of the 60 ICIs rechallenge studies included in this analysis, 34 reported all grades of irAEs, 16 reported the occurrence of grade 1-2 irAEs, and 42 reported the prevalence of grade ≥ 3 irAEs. On meta-analysis, the overall incidence of all grades of irAEs was 57.1% (95% CI: 47.3, 66.7), 33.5% (95% CI: 22.6, 45.4) for grade 1-2 irAEs, and 16.7% (95% CI: 11.8, 22.2) for grade ≥ 3 irAEs (**Figures 6A, B**). Regarding the incidence of grade ≥ 3 irAEs after retreatment with ICIs, further subgroup analyses were performed on those with three or more relevant studies of the different tumor histological types. The incidence of grade ≥ 3 irAEs was higher in melanoma and renal cell carcinoma with 20.1% (95% CI: 12.3, 29.0) and 27.1% (95% CI: 12.1, 45.3), respectively. Whereas the incidence of grade ≥ 3 irAEs in lung cancer was only 7.6% (95% CI: 3.8, 12.4) (**Figure 7A**). We went on to explore the effect of different regimens on rechallenge adverse reactions to ICIs. The results demonstrated that the incidence of grade ≥ 3 irAEs was 26.6% (95% CI: 10.2, 46.7) in those using anti-CTLA-4 antibody alone, which was higher than that in those using a combination of anti-CTLA-4 and anti-PD-(L)1 antibody [25.4%, (95% CI: 17.8, 33.7)], and in those using anti-PD-(L)1 antibody alone [8.1%, (95% CI: 5.0, 11.7)] (**Figure 7B**).

### 3.4 Publication bias

**Figure 8:**
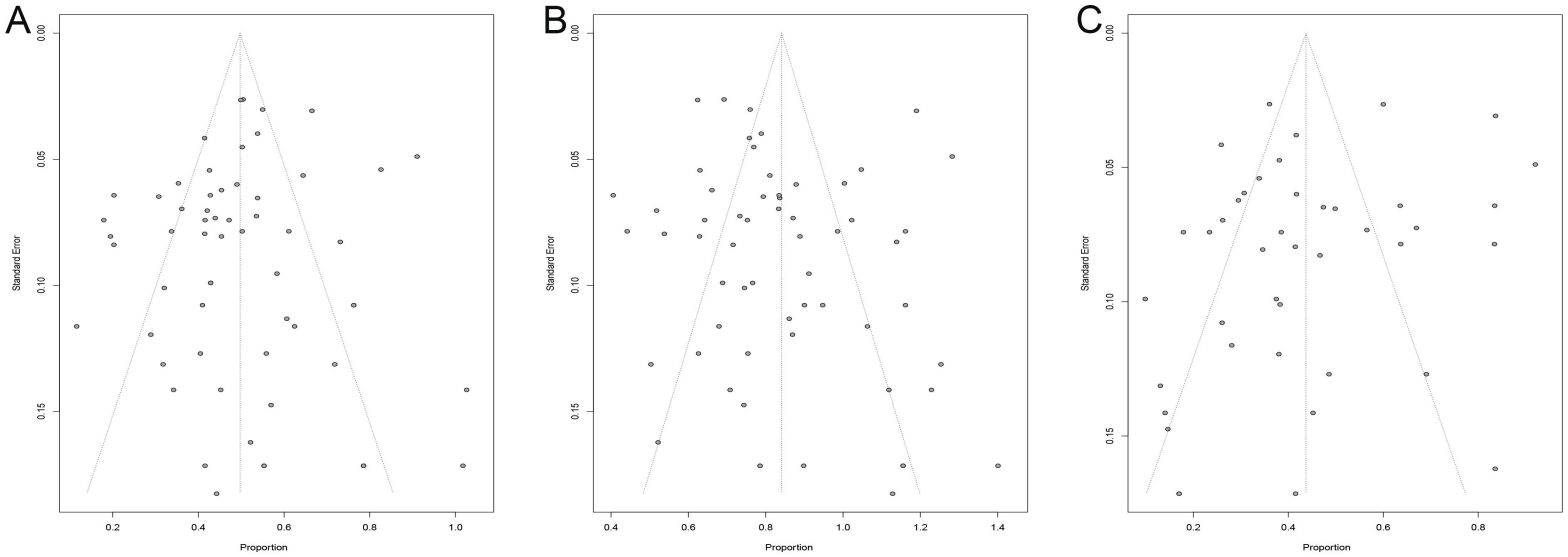
Funnel plot. **(A)** funnel plot for ORR; **(B)** funnel plot for DCR; **(C)** funnel plot for irAEs. ORR, overall objective response rate; DCR, disease control rate; irAEs, immune-related adverse events.

Our research focused on exploring the efficacy and safety of ICIs rechallenge in advanced solid tumors, therefore publication bias analysis was performed with ORR, DCR, and incidence of grade ≥ 3 irAEs as endpoints. We analyzed the heterogeneity of these included studies and obtained asymmetric funnel plots, which suggests that there is heterogeneity in these studies. After Egger’s test, the p values for ORR, DCR, and incidence of grade ≥ 3 irAEs were 0.41, 0.32, and 0.10, respectively, all greater than 0.05, indicating that these studies had low publication bias (**Figure 8**).

## 4 Discussion

In this study, we systematically searched the literature regarding the reintroduction of ICIs therapy in patients with advanced or refractory solid tumors after interruption of ICIs therapy and performed a meta-analysis, which revealed that the ORR and DCR of reintroduction of ICIs were 21.6% and 55.8%, respectively. This study also demonstrated that the incidence of grade ≥ 3 irAEs at reintroduction of ICIs was 16.7%, which was not significantly higher than that at previous ICIs treatment as reported in previous literature (18–21). In this study, subgroup analyses were performed based on different tumor histological types and different regimens at ICIs reuse. The efficacy of ICIs rechallenge was found to be more favorable in melanoma and renal cell carcinoma than in lung cancer, with ORRs of 24.3%, 30.9%, and 10.1%, respectively. In patients who have been treated with single-target ICIs, retreatment with a combination of ICIs directing different targets presents better outcomes than in patients who continue to use a single agent, but have a higher incidence of irAEs. When resistance or progression occurred after prior treatment with either anti-PD-(L)1 or anti-CTLA-4 antibodies and ICIs was reintroduced, patients who chose anti-PD-(L)1 antibodies had a higher ORR than those who used anti-CTLA-4 antibodies. This study confirms that re-administration of ICIs controls the progression of advanced or refractory solid tumors with a relatively low incidence of irAEs, which provides a rationale for ICIs rechallenge in patients with advanced or refractory solid tumors who have been previously treated with ICIs.

A total of 60 studies were included in this meta-analysis, of which 2 were phase III RCTs and the rest were retrospective or observational. We found that patients with advanced or refractory solid tumors had an ORR of 21.6% for reintroduction of ICIs after interruption of ICIs, similar to the 21.8% reported by Inno A et al. (22). The meta-analysis by Inno et al. in 2021 included 49 studies, fewer than those incorporated in our analysis (22). We did not assess OS and progress free survival (PFS) in this study given that these patients have been administered multiple lines of systemic therapy in the past, and OS and PFS are affected by a few confounding factors such as previous treatment, general performance status and comorbidities. Currently, there are inconsistent reports on the efficacy of reuse of ICIs after recurrence or progression in patients with solid tumors previously treated with ICIs. A multicenter retrospective study by Nardin et al. (23) included 85 patients with advanced melanoma who achieved remission or stable after a single previous course of ICIs treatment and who were retreated with ICIs after disease progression, with an ORR of 54% and a DCR of 75%. This result suggests that patients who respond well to previous ICIs therapy have favorable outcomes when treated again with ICIs. In another multicenter retrospective study of non-small cell lung cancer (NSCLC), 144 patients who had been treated with anti-PD(L)1 antibodies and who had interrupted their ICIs therapy due to toxicity or disease progression had an ORR and DCR of 15.9% and 47.2%, respectively, after re-administration of anti-PD(L)1 antibody (7). These two studies employed the same regimen as previous ICIs therapy for most patients when reintroducing ICIs treatment, but outcomes varied significantly. Twenty-one patients in a study of advanced hepatocellular carcinoma experienced disease progression after previous ICIs treatment, and 11 (52%) patients presented partial response (PR) or stable disease (SD) on retreatment with different ICIs regimens (24). The study concluded that even after a poor response to previous ICIs treatment, patients with advanced hepatocellular carcinoma remain responsive to a different ICIs regimen in the rechallenge. The CONTACT-03 study randomized 522 patients with advanced or metastatic renal cell carcinoma into the anti-programmed death-ligand 1 (PD-L1) monoclonal inhibitor combined with tyrosine kinase inhibitor (TKI) group and the TKI alone group, and found that the ORRs of the two groups were 38.0% and 41.7%, respectively (25). It indicated that switching to TKI after progression with previous ICIs treatment could control tumor progression, but the addition of an anti-PD-L1 monoclonal inhibitor did not improve the efficacy. The above findings indicate a wide variability in response to re-administration of ICIs to different types of solid tumors. To further explore which type of solid tumors benefits more from ICIs retreatment, we performed subgroup analyses and found that renal cell carcinoma presented superior efficacy with an ORR of 30.9%, which was higher than that of melanoma (24.3%) and NSCLC (10.1%). The differences in outcome may be related to the expression level of PD-L1 in different tumors. Analyzing 60 patients with advanced gastric cancer, Xin et al. found that patients with high PD-L1 expression in tumor tissues had a better outcome of re-treatment with anti-PD-1 antibodies, with an ORR of 22.7%, compared to an ORR of 9.1% for those with low expression (26). Fujita K’s study of NSCLC patients undergoing immune rechallenge demonstrated that those with high PD-L1 expression (tumor proportion score ≥ 80%) achieved PR and SD (27). In addition to PD-L1 expression levels, two studies of NSCLC patients with ICIs retreatment confirmed that Eastern Cooperative Oncology Group Performance Score (ECOG-PS) was an independent prognostic factor affecting PFS or OS (28, 29). One of the studies also found that when compared with body mass index (BMI) ≤ 20, patients with BMI > 20 had more favorable PFS (HR 0.43, p = 0.036) (28). A national multicenter retrospective study from France identified that receiving systemic therapy (including chemotherapy, targeted therapy, or a clinical trial) between initial treatment and rechallenge with ICIs lowered the ORR and PFS during rechallenge in patients with melanoma (23). These results suggest that the efficacy of rechallenge with ICIs is influenced by tumor type, prior and rechallenge regimens for ICIs, PD-L1 level of expression, and the patient’s global status.

Patients with advanced or recurrent solid tumors may still benefit from the reuse of ICIs. To explore the optimal treatment regimen for ICIs rechallenge, we further performed subgroup analyses based on different regimens for reuse of ICIs. The study showed that in patients who have been treated with single-target ICIs, retreatment with a combination of ICIs directing different targets presents better outcomes than in patients who continue to use a single agent, while those who remained on single-target ICIs treatment had a higher ORR with anti-PD-(L)1 antibodies than with anti-CTLA-4 antibodies. The results are consistent with those reported in the KEYNOTE-002 study, a melanoma study of repeat ICIs use (12). For the single agent re-use, most studies included in this systematic review analysis described only the drugs and efficacy of re-used ICIs, but did not address the exact drugs, their ORRs, and DCRs upon initial immune-therapy. One study of NSCLC using PD-1 antibody for initial treatment had an ORR and DCR of 21.1% and 63.2%, respectively, and re-immunotherapy with a PD-L1 antibody had an ORR of 2.6% and a DCR of 34.2% (29). Two studies on melanoma switched from a PD-1 antibody to a CTLA-4 antibody during immunotherapy rechallenge. These two studies had an initial ORR of 20% and 44.4% and a DCR of 55.5% and 57.5%, respectively, and an ORR of 10.0% and 22.2% and a DCR of 17.5% and 22.2% at rechallenge, respectively (13, 30). Only the study by Bowyer et al. on 40 metastatic melanomas described 2 patients developing grade ≥ 3 irAEs both at the time of initial immunotherapy and at the time of re-treatment (13), whereas none of the other studies documented the incidence of irAEs at initial use of ICIs. Because fewer than 3 studies were included in each of the above single-agent replacement regimens for immunotherapy rechallenge, we did not conduct a meta-analysis of the efficacy and toxicity of the regimens, which will be analyzed in the future with the inclusion of more studies. In addition to the above regimens, regimens such as ICIs in combination with chemotherapy, or in combination with anti-vascular endothelial growth factor (VEGF) antibodies were also included in the studies for the present analysis but could not be meta-analyzed due to the paucity of literature. In the real world, clinical trials exploring new therapeutic modalities, such as combining ICIs with different targets, combining ICIs with anti-angiogenic agents, combining ICIs with chemotherapy and combining ICIs with radiotherapy, are ongoing with a view to improving the immunosuppressive state of the tumor microenvironment through different mechanisms, thereby enhancing the treatment efficacy and providing new insight into the rechallenging ICIs.

In addition to the efficacy of ICIs reuse in advanced or refractory solid tumors, toxicities and side effects affect patients’ quality of life and tumor outcomes. The present study found a 16.7% incidence of grade ≥ 3 irAEs in these patients reintroduced to ICIs, which is not significantly higher than the 17.3% to 31% incidence reported in the literature for previous ICIs (31, 32), but is higher than the incidence of grade ≥ 3 irAEs in rechallenge with ICIs from the meta-analysis by Zhao et al. (11.7%) (33). The higher incidence of grade ≥ 3 irAEs in this study is probably related to the inclusion of more literature and the use of more ICIs rechallenge regimens in the literature. Multiple rechallenge regimens based on ICIs may enhance efficacy, but also lead to a higher incidence of adverse events (34, 35). Whether the toxicity at re-use of ICIs is influenced by different tumor histological types and different regimens is also a concern. In our study, a subgroup analysis of the incidence of adverse events according to different tumor histological types revealed that grade ≥ 3 irAEs were found in 20.1%, 27.1%, and 7.6% of patients with melanoma, renal cell carcinoma, and NSCLC, respectively, at re-introduction of ICIs. The incidence of grade ≥ 3 irAEs was 26.6% in patients using anti-CTLA-4 antibodies during immune rechallenge, higher than that in patients using a combination of anti-CTLA-4 antibodies and anti-PD-(L)1 antibodies (25.4%) and anti-PD-(L)1 antibodies alone (8.1%), which is thought to be related to the fact that CTLA-4 and PD-(L)1 function at different stage in the immune response. CTLA-4 has been shown to affect T-cell proliferation and activation in the early phase of the immune response, which occurs predominantly in lymph nodes, whereas PD-(L)1 inhibits T-cell killing in the late phase of the immune response, which occurs mainly in peripheral tissues, leading to a different incidence of adverse events (36). Higher incidence of grade ≥ 3 irAEs in patients retreated with anti-CTLA-4 antibody and anti-PD-(L)1 antibody combination therapy was concluded in our study, which may be attributed to the simultaneous action of anti-CTLA-4 and anti-PD(L)-1 antibodies on T cells, leading to T cell proliferation and activation, as well as the increased affinity of T cells for cross-antigens expressed in normal tissues, which can release a large number of inflammatory factors to attack normal tissues, leading to the development of irAEs (37–39). The specific type of grade ≥ 3 irAEs reported varied across studies. With respect to the reintroduction of ICIs in patients with melanoma and renal cell carcinoma, the most common serious irAEs included gastrointestinal symptoms such as diarrhea, skin rashes, and hepatic dysfunction, whereas immune pneumonitis and gastrointestinal symptoms were most common in patients with NSCLC (18–20, 40–47). Therefore, when ICIs are re-administered in advanced or refractory solid tumors, the potential toxicities of ICIs need to be fully assessed and closely monitored. There is no consensus on whether patients who have discontinued ICIs due to severe adverse effects from previous ICIs therapy can be re-treated with ICIs. Clinicians need to fully assess the risks and benefits of ICIs rechallenge for each patient and select regimens carefully without increasing toxicity.

This study systematically analyzed the efficacy and toxicities of re-administration of ICIs in advanced or refractory solid tumors, providing evidence for clinical decision-making, but with some limitations. Firstly, there was significant heterogeneity in the studies included in this analysis, considered to be related to the wide variation in sample sizes among the studies and the lack of information in some studies about the specific regimen of prior therapy and the dose, interval and number of cycles of ICIs to be reintroduced. Secondly, most studies did not provide PD-L1 expression in tumor tissues, limiting further analysis in this study to predict the efficacy of ICIs rechallenge. Thirdly, most of the studies included in this analysis were retrospective observational studies that lacked controls and lacked outcomes after long-term follow-up, and thus PFS and OS were not analyzed.

## 5 Conclusion

Rechallenge with ICIs can be recommended for patients with an advanced or refractory solid tumor experiences relapse or progression after prior ICIs therapy, as this regimen offers a favorable ORR and DCR. For patients who have been treated with single-target ICIs, retreatment with a combination of ICIs against different targets results in better efficacy than continued single-agent therapy, but with a higher incidence of adverse effects. These findings may guide clinical decision-making during ICIs rechallenge, particularly with regard to the choice of whether to combine different targets or single-target therapy, while alerting clinicians to closely monitor for related adverse effects. As the studies included in this analysis were mostly retrospective, this conclusion needs to be further confirmed by prospective studies with large samples and multiple centers.

## Data Availability

The original contributions presented in the study are included in the article/**Supplementary Material**. Further inquiries can be directed to the corresponding author.

## Supplementary material

Supplementary Table 1 Search strategy in PubMed.

Supplementary Table 2 Characteristics and quality assessment of the studies included in the meta-analysis.
