# Supplementary material for: Efficacy and safety of immune checkpoint inhibitors rechallenge in advanced solid tumors: a systematic review and meta-analysis
Source: Front Oncol. 2024 Dec 12;14:1475502. doi: 10.3389/fonc.2024.1475502 (PMC11669585; doi:10.3389/fonc.2024.1475502)
Supplement: Supplementary Table 1 — Search strategy in PubMed. [file Table1.docx]

Supplementary Material

# Supplementary Tables

**Table S1: Search strategy in PubMed.**

| 1# | ((((((((Neoplasms[MeSH Terms]) OR (Tumor*[Title/Abstract])) OR (Neoplasm[Title/Abstract])) OR (Neoplasia*[Title/Abstract])) OR (Cancer*[Title/Abstract])) OR (Malignant Neoplasm*[Title/Abstract])) OR (Malignancy[Title/Abstract])) OR (Malignancies[Title/Abstract])) OR (Carcinoma*[Title/Abstract]) |
| --- | --- |
| 2# | (((reuse[Title/Abstract]) OR (rechallenge[Title/Abstract])) OR (restart[Title/Abstract])) OR (retreatment[Title/Abstract]) |
| 3# | ((((((((((Immune Checkpoint Inhibitors[MeSH Terms]) OR (Immun*[Title/Abstract])) OR (Programmed Cell Death 1 Receptor[Title/Abstract])) OR (PD-1[Title/Abstract])) OR (PD-L1[Title/Abstract])) OR (immunotherapy[Title/Abstract])) OR (CTLA-4[Title/Abstract])) OR (ICI*[ Title/Abstract])) OR (Nivolumab[Title/Abstract])) OR (pembrolizumab[Title/Abstract])) OR (Ipilimumab[Title/Abstract]) |
| 4# | 1# AND 2# AND 3# |
