# Supplementary material for: Efficacy and safety of immune checkpoint inhibitors rechallenge in advanced solid tumors: a systematic review and meta-analysis
Source: Front Oncol. 2024 Dec 12;14:1475502. doi: 10.3389/fonc.2024.1475502 (PMC11669585; doi:10.3389/fonc.2024.1475502)
Supplement: Supplementary Table 2 — Characteristics and quality assessment of the studies included in the meta-analysis. [file Table2.docx]

Supplementary Material

# Supplementary Tables

**Table S2: characteristics of selected studies in the meta-analysis**

| **Reference** | **Sample size** | **Types of solid tumors** | **Type of study** | **Initial ICI type (%)** | **Rechallenge ICI type (%)** | **Cause of interruption (%)** | **ORR (%)** | **DCR (%)** | **All grade irAEs**  **Rechallenge (%)** | **Grade ≥3 irAEs Rechallenge (%)** | **Quality**  **(Mirror score)** |
| --- | --- | --- | --- | --- | --- | --- | --- | --- | --- | --- | --- |
| Robert C/2013  (1) | 38 | Melanoma | Respective | Ipi ± gp-100 | Ipi ± gp-100 | PD (100) | 18.4 | 60.5 | 57.9 | 10.5 | 16 |
| Robert C/2014  (2) | 173 | Melanoma | Phase 1 trial | Ipi | Pem (2mg/kg or 10mg/kg) | PD (100) | 26.1 | 50.3 | 82.1 | 16.2 | 22 |
| Chiarion-Sileni/2014  (3) | 51 | Melanoma | Respective | Ipi | Ipi | PD (100) | 11.7 | 54.9 | 21.6 | 5.9 | 8 |
| Lebbé C/2014  (4) | 122 | Melanoma | Respective | Ipi | Ipi | PD (100) | 23.0 | 48.4 | 64.0 | 13.5 | 10 |
| Ribas A/2015  (5) | 361 | Melanoma | Randomized controlled phase 2 trial | Ipi | Pem (2mg/kg or 10mg/kg) | PD (100) | 23.3 | 40.7 | 58.3 | 12.3 | 18 |
| Larkin J/2018  (6) | 272 | Melanoma | RCT | Ipi | Niv | PD (100) | 27.2 | 47.4 | / | / | 18 |
| Bowyer S/2016  (7) | 40 | Melanoma | Respective | Pem; Niv | Ipi | PD (100) | 10.0 | 17.5 | / | 35.0 | 8 |
| Zimmer L/2017  (8) | 84 | Melanoma | Respective | Anti-PD-1 | Ipi (56.0); Ipi + Niv（44.0） | PD (100) | 16.7 | 34.5 | / | / | 8 |
| Betof WA/2020  (9) | 78 | Melanoma | Respective | Pem; Niv | Anti-PD-1(43.6); Anti-PD-1 + Ipi (56.4) | PD (100) | 35.9 | 52.3 | / | / | 10 |
| Amode R/2017  (10) | 39 | Melanoma | Respective | Ipi | Pem; Niv | Toxicity (100) | 15.4 | 25.6 | 48.7 | 15.4 | 10 |
| Jacobsoone-Ulrich A/2016  (11) | 8 | Melanoma | Respective | Pem(75)；Niv (25) | Ipi (100) | PD (100) | 50 | 50 | 37.5 | 12.5 | 8 |
| Aya F/2016  (12) | 9 | Melanoma | Respective | Pem (77.8)；Niv (22.2) | Ipi (100) | PD (100) | 22.2 | 22.2 | 55.6 | 55.6 | 9 |
| Nomura M/2017  (13) | 8 | Melanoma | Respective | Niv (100) | Niv (100) | PD (100) | 25 | 62.5 | 37.5 | 0 | 9 |
| Blasig H /2017  (14) | 8 | Melanoma | Respective | Pem(25)；Niv (75) | Pem (100) | PD (62.5); Toxicity (12.5); Other (25) | 12.5 | 50.0 | / | / | 8 |
| Fujisawa Y/2018  (15) | 60 | Melanoma | Respective | Niv (100) | Ipi (100) | PD (93); Toxicity (7) | 3.3 | 15.0 | 78.3 | 55.0 | 10 |
| Jansen YJL/2019  (16) | 19 | Melanoma | Respective | Anti-PD-1 (100) | Pem (73.7); Niv (21.1) | PD (100) | 31.6 | 57.9 | / | / | 9 |
| Whitman E /2020  (17) | 21 | Melanoma | Respective | Pem (47.6); Niv (52.4) | Pem (38.1); Niv (61.8) | Adverse effect (35);  Other noncancer–related issue(s) (25);  Drug holiday (15);  Cancer-related symptom(s) not related to therapy (10); Other (15) | 47.6 | 61.9 | / | / | 9 |
| Pires da Silva I/ 2021  (18) | 355 | Melanoma | Respective | Anti-PD-(L)1 (100) | Ipi (45.6); Anti-PD-1 + Ipi (54.4) | PD (100) | 22.8 | 34.1 | 80.0 | 31.8 | 10 |
| Asher N/ 2021  (19) | 21 | Melanoma | Respective | Anti-PD-1 (81); Ipi + Niv (19) | Anti-PD-1(90.5); Ipi + Niv (4.8); Ipi (4.8) | Toxicity; Clinical dicision | 47.6 | 66.7 | / | / | 9 |
| Pokorny R / 2021  (20) | 8 | Melanoma | Respective | Anti-PD-1 | Anti-PD-1 | Clinical dicision | 50.0 | 87.5 | / | / | 8 |
| Persa OD /2021  (21) | 20 | Melanoma | Respective | Anti-PD-1; Anti-PD-1 + anti-CTLA-4 | Anti-PD-1; Anti-CTLA-4; Anti-PD-1 + anti-CTLA-4 | PD | 16.0 | 24.0 | / | / | 9 |
| Hepner A / 2021  (22) | 47 | Melanoma | Respective | Ipi + Niv (83); Pem-based (15); Ipi alone (2) | Ipi + Niv (83); Pem-based (2); Ipi alone (13); Other (2) | PD | 25.5 | 44.7 | 46.8 | 38.3 | 8 |
| van Zeijl/ 2022  (23) | 27 | Melanoma | Respective | Anti-PD-1 | Anti-PD-1 | Toxicity; PD; Clinical dicision | 29.6 | 63.0 | / | / | 10 |
| Nardin C /2023  (24) | 85 | Melanoma | Respective | Anti-PD-1(80)  (Pem48, Niv32);  Ipi + Niv (8); Ipi (12) | Anti-PD-1; Ipi; Ipi + Niv | PD | 51.1 | 75.3 | 32.9 | 10.6 | 10 |
| Gobbini/2020  (25) | 144 | NSCLC | Respective | Anti-PD-(L)1 | Anti-PD-(L)1 | PD (40); Toxicity (40);  Clinical dicision (20) | 15.9 | 47.2 | / | 6.3 | 12 |
| Herbst RS/2020  (26) | 14 | NSCLC | Respective | Pem | Pem | Discontinued (35.7); PD (28.5) | 42.9 | 92.9 | / | / | 14 |
| Niki M/2018  (27) | 11 | NSCLC | Respective | Niv (100) | Niv (90.9); Pem (9.1) | / | 27.3 | 45.5 | 81.8 | 0 | 8 |
| Fujita K/2018  (28) | 12 | NSCLC | Respective | Niv | Pem | PD (100) | 8.3 | 41.7 | / | 12.5 | 8 |
| Fujita K/2019  (29) | 18 | NSCLC | Respective | Pem (38.9)；Niv (44.4)；Pem + Niv (16.7) | Atez (100) | PD (100) | 0 | 38.9 | / | / | 8 |
| Katayama Y/2019  (30) | 35 | NSCLC | Respective | Atez (11.4)；Niv (54.3)；Pem (34.3) | Atez (65.7)；Niv (14.3)；Pem (20.0) | PD (100) | 2.9 | 42.9 | / | / | 10 |
| Watanabe H/2019  (31) | 14 | NSCLC | Respective | Atez (14.3)；Niv (78.6)；Pem (7.1) | Niv (64.3)；Pem (35.7) | PD (100) | 7.1 | 21.4 | 35.7 | 0 | 8 |
| Mouri A/2019  (32) | 21 | NSCLC | Respective | Niv | Niv | Toxicity (100) | 14.3 | 85.7 | 71.4 | 4.8 | 10 |
| Sternschuss M/2020  (33) | 15 | NSCLC | Respective | Atez (6.7); Pem (20); Niv (73.3) | Ipi + anti-PD-1 | PD (100) | 13.3 | 33.3 | 40.0 | 20.0 | 9 |
| Kitagawa S/2020  (34) | 17 | NSCLC | Respective | Anti-PD-(L)1 | Anti-PD-(L)1 | PD (58.9); Toxicity (41.1) | 5.9 | 58.8 | 64.7 | 11.8 | 8 |
| Furuya N /2021  (35) | 38 | NSCLC | Respective | Anti-PD-1 | Atez | PD (100) | 2.6 | 34.2 | / | / | 9 |
| Takahara Y/2022  (36) | 24 | NSCLC | Respective | Anti-PD-(L)1 | Anti-PD-(L)1 | PD (70.8); Other (29.2) | 8.3 | 45.8 | 16.7 | 12.5 | 8 |
| Yang J/2022  (37) | 45 | Advanced lung cancer | Respective | Anti-PD-1 (82.2); Anti-PD-L1 (17.8) | Anti-PD-(L)1 | PD (11.1); Toxicity (88.9) | 2.2 | 73.3 | 20.0 | 2.2 | 12 |
| Akamatsu H/2022  (38) | 59 | NSCLC | Phase 2 trial | Anti-PD-(L)1 alone (91); ICI + cytotoxic chemotherapy (9) | Niv | NA | 8.5 | 50.8 | / | 20.3 | 10 |
| Bilger G/al. 2022  (39) | 7 | NSCLC | Respective | ICI | ICI | NA | 14.3 | 85.7 | / | / | 8 |
| Xu Z/2022  (40) | 40 | NSCLC | Respective | Anti-PD-1 alone (25); Anti-PD-1+chemotherapy (52.5); Anti-PD-1+angiogenesis inhibitor (12.5); Anti-PD-1+chemotherapy+angiogenesis inhibitor (10) | Anti-PD-1 alone (7.5); Anti-PD-(L)1+chemotherapy (42.5); Anti-PD-1+angiogenesis inhibitor (25); Anti-PD-1+chemotherapy+angiogenesis inhibitor (25) | PD (100) | 22.5 | 85.0 | / | / | 10 |
| Torasawa M/2023  (41) | 64 | NSCLC | Respective | Anti-PD-(L)1; ICI + chemotherapy | Anti-PD-(L)1(93.7); Anti-PD-1 + anti-CTLA-4(6.3) | PD (46.9); Clinical decisions and irAEs (53.1) | 18.8 | 37.5 | 28.1 | 7.8 | 9 |
| Gul/2020  (42) | 45 | RCC | Respective | Anti-PD-1 | Ipi + Niv | PD; Toxicity; Other | 20.0 | 35.6 | 64.4 | 13.3 | 10 |
| Alaiwi/2020  (43) | 36 | RCC | Respective | ICI alone；ICI + anti-VEGF；Dual ICI | ICI alone；ICI + anti-VEGF；Dual ICI | Toxicity (100) | 44.4 | 83.3 | 50.0 | 19.4 | 10 |
| Ravi P/2020  (44) | 69 | RCC | Respective | ICI alone (39); Dual ICI (13); ICI + TT (42); ICI + chemotherapy (3); ICI + investigational agent (3) | Single ICI (38); Dual ICI (32); ICI + TT (19); ICI + chemotherapy (1); ICI + investigational agent (10) | PD(72); Toxicity(23);Other（3） | 21.7 | 59.4 | 44.9 | 15.9 | 11 |
| Lee CH/2021  (45) | 104 | RCC | Phase 1/2 trial | Anti-PD-(L)1 | Pem + lenvatinib | NA | 62.5 | 92.3 | 99.0 | 35.6 | 9 |
| Choueiri TK/2022  (46) | 46 | RCC | Respective | Anti-PD-(L)1; Anti-CTLA-4 | Niv + Ipi | PD (100) | 17.4 | 58.7 | 78.2 | 28.3 | 9 |
| Vauchier C /2022  (47) | 45 | RCC | Respective | ICI alone (82); Niv + Ipi (11); ICI + TT (7) | Niv (93); Niv + Ipi (7) | PD (94)；Toxicity (3)；Clinical decision (3) | 15.6 | 46.7 | 4.4 | 4.4 | 9 |
| Pal SK/2023  (48) | 263 | RCC | RCT | Ipi + Niv; Anti-PD-(L)1 | Ate + cabozantinib | PD (100) | 38.0 | 86.3 | 95.8 | 55.1 | 18 |
| Bernard T/2018  (49) | 8 | various | Respective | Anti-PD-(L)1 | Anti-PD-(L)1 | PD (100) | 25.0 | 100 | / | 0 | 10 |
| Simonaggio A/2019  (50) | 40 | various | Respective | Anti-PD-(L)1；Anti-CTLA-4; Anti–PD-1+anti-CTLA-4; ICI + chemotherapy | Anti-PD-(L)1 (77.5); Anti-PD-1+anti-CTLA-4 (10); Others (12.5) | Toxicity (100) | 32.5 | 70.0 | 57.5 | 55.0 | 10 |
| Sheth S/2020  (51) | 70 | various | Respective | Dur | Dur | Disease control; Other clinical benefit | 11.4 | 71.4 | 40.0 | 8.6 | 9 |
| Wong JSL/2020  (52) | 25 | HCC | Respective | Niv (76); Pem (20); Atez + bevacizumab (4) | Ipi + anti-PD-1 | PD (100) | 16.0 | 40.0 | 52.0 | 12.0 | 9 |
| Scheiner B/2022  (53) | 58 | HCC | Respective | ICI alone (44.8); Dual ICI (1.7); ICI + TT/anti-VEGF (53.5) | ICI monotherapy (6.9); Dual ICI combination (20.7); ICI + TT/anti-VEGF (72.4) | PD (89.7)；Toxicity (6.9)；Other (3.4) | 25.9 | 55.2 | 48.3 | 22.4 | 10 |
| Ito K/2022  (54) | 12 | urothelial carcinoma | Respective | Pem | Pem | Toxicity; Disease control; Comorbidities | 75.0 | 91.7 | / | / | 8 |
| Makrakis D/2023  (55) | 25 | urothelial carcinoma | Respective | Anti-PD-(L)1 alone; Dual anti-PD-(L)1 | Anti-PD-(L)1 alone; Dual anti-PD-(L)1 | NA | 16.0 | 48.0 | / | 0 | 8 |
| Xinxin Z/2023  (56) | 60 | GC | Respective | Anti-PD-1; Anti-PD-1 + chemotherapy; Anti-PD-1 + anti-VEGF | Anti-PD-1; Anti-PD-1 + chemotherapy; Anti-PD-1 + anti-VEGF | NA | 16.7 | 55.0 | / | 35.0 | 11 |
| Li G/2023  (57) | 15 | CC | Respective | ICI alone (26.7); ICI + chemotherapy (73.3) | Cam + paclitaxel + apatinib | NA | 26.7 | 46.7 | 80.0 | 40.0 | 9 |
| Wakasugi T/2022  (58) | 12 | Head and neck cancer | Respective | Niv (91.7); Pem (8.3) | Niv (100) | PD (100) | 16.7 | 83.3 | / | 0 | 9 |
| Ding X/2023  (59) | 18 | NPC | Phase 2 trial | Anti-PD-1 | Cam + famitinib | PD (100) | 33.3 | 77.8 | 100 | 5.6 | 12 |
| Weppler AM/2023  (60) | 8 | Merkel cell carcinoma | Respective | Anti-PD-(L)1 | Anti-PD-(L)1 | Toxicity; Clinical decision; | 75.0 | 87.5 | / | 0 | 9 |

ICI: immune checkpoint inhibitor; ORR, overall response rate; irAEs, immune-related adverse events; RCT, Randomized controlled trial; NSCLC, non-small-cell lung cancer; RCC, renal cell carcinoma; HCC, hepatocellular carcinoma; GC, gastric carcinoma; CC, cervical cancer, NPC, nasopharyngeal carcinoma; PD-(L)1, programmed death-(ligand) 1; CTLA-4, cytotoxic T-lymphocyte antigen 4; Ipi, ipilimumab; Pem, pembrolizumab; Niv, nivolumab; Dur, Durvalumab; Atez, atezolizumab; Cam, camrelizumab, TT, target therapy; gp-100, the peptide gp100 vaccine:209-217(210M); VEGF, vascular endothelial growth factor; PD, progression disease; NA: not available.

**References**

1. Robert C, Schadendorf D, Messina M, Hodi FS, O'Day S; MDX010-20 investigators. Efficacy and Safety of Retreatment with Ipilimumab in Patients with Pretreated Advanced Melanoma Who Progressed after Initially Achieving Disease Control. Clinical Cancer Research (2013) 19:2232–2239. doi: 10.1158/1078-0432.CCR-12-3080.
2. Robert C, Ribas A, Wolchok JD, Hodi FS, Hamid O, Kefford R, *et al.* Anti-programmed-death-receptor-1 treatment with pembrolizumab in ipilimumab-refractory advanced melanoma: a randomised dose-comparison cohort of a phase 1 trial. Lancet (2014) 384:1109–1117. doi: 10.1016/S0140-6736(14)60958-2.
3. Chiarion-Sileni V, Pigozzo J, Ascierto PA, Simeone E, Maio M, Calabrò L, *et al.* Ipilimumab retreatment in patients with pretreated advanced melanoma: the expanded access programme in Italy. Br J Cancer (2014) 110:1721–1726. doi: 10.1038/bjc.2014.126.
4. Lebbé C, Weber JS, Maio M, Neyns B, Harmankaya K, Hamid O, *et al.* Survival follow-up and ipilimumab retreatment of patients with advanced melanoma who received ipilimumab in prior phase II studies. Ann Oncol (2014) 5:2277–2284. doi: 10.1093/annonc/mdu441.
5. Ribas A, Puzanov I, Dummer R, Schadendorf D, Hamid O, Robert C, *et al.* Pembrolizumab versus investigator-choice chemotherapy for ipilimumab-refractory melanoma (KEYNOTE-002): a randomised, controlled, phase 2 trial. The Lancet Oncology (2015) 16:908–918. doi: 10.1016/S1470-2045(15)00083-2.
6. Larkin J, Minor D, D'Angelo S, Neyns B, Smylie M, Miller WH Jr, *et al.* Overall Survival in Patients With Advanced Melanoma Who Received Nivolumab Versus Investigator’s Choice Chemotherapy in CheckMate 037: A Randomized, Controlled, Open-Label Phase III Trial. J Clin Oncol (2018) 36:383–390. doi: 10.1200/JCO.2016.71.8023.
7. Bowyer S, Prithviraj P, Lorigan P, Larkin J, McArthur G, Atkinson V, *et al.* Efficacy and toxicity of treatment with the anti-CTLA-4 antibody ipilimumab in patients with metastatic melanoma after prior anti-PD-1 therapy. Br J Cancer (2016) 114:1084–1089. doi: 10.1038/bjc.2016.107.
8. Zimmer L, Apuri S, Eroglu Z, Kottschade LA, Forschner A, Gutzmer R, *et al*. Ipilimumab alone or in combination with nivolumab after progression on anti-PD-1 therapy in advanced melanoma. European Journal of Cancer (2017) 75:47–55. doi: 10.1016/j.ejca.2017.01.009.
9. Betof Warner A, Palmer JS, Shoushtari AN, Goldman DA, Panageas KS, Hayes SA, *et al.* Long-Term Outcomes and Responses to Retreatment in Patients With Melanoma Treated With PD-1 Blockade. JCO (2020) 38:1655–1663. doi: 10.1200/JCO.19.01464.
10. Amode R, Baroudjian B, Kowal A, Jebali M, Allayous C, Bagot M, *et al.* Anti-programmed cell death protein 1 tolerance and efficacy after ipilimumab immunotherapy: observational study of 39 patients. Melanoma Res (2017) 27:110–115. doi: 10.1097/CMR.0000000000000313.
11. Jacobsoone-Ulrich A, Jamme P, Alkeraye S, Dzwiniel V, Faure E, Templier C, *et al.* Ipilimumab in anti-PD1 refractory metastatic melanoma: a report of eight cases. Melanoma Res (2016) 26:153–156. doi: 10.1097/CMR.0000000000000221.
12. Aya F, Gaba L, Victoria I, Fernández-Martínez A, Tosca M, Prat A, *et al*. Ipilimumab after progression on anti-PD-1 treatment in advanced melanoma. Future Oncol (2016) 12:2683–2688. doi: 10.2217/fon-2016-0037.
13. Nomura M, Otsuka A, Kondo T, Nagai H, Nonomura Y, Kaku Y, *et al.* Efficacy and safety of retreatment with nivolumab in metastatic melanoma patients previously treated with nivolumab. Cancer Chemother Pharmacol (2017) 80:999–1004. doi: 10.1007/s00280-017-3444-0.
14. Blasig H, Bender C, Hassel JC, Eigentler TK, Sachse MM, Hiernickel J, *et al.* Reinduction of PD1-inhibitor therapy: first experience in eight patients with metastatic melanoma. Melanoma Research (2017) 27:321–325. doi: 10.1097/CMR.0000000000000341.
15. Fujisawa Y, Yoshino K, Otsuka A, Funakoshi T, Uchi H, Fujimura T, *et al.* Retrospective study of advanced melanoma patients treated with ipilimumab after nivolumab: Analysis of 60 Japanese patients. J Dermatol Sci (2018) 89:60–66. doi: 10.1016/j.jdermsci.2017.10.009.
16. Jansen YJL, Rozeman EA, Mason R, Goldinger SM, Geukes Foppen MH, Hoejberg L, *et al.* Discontinuation of anti-PD-1 antibody therapy in the absence of disease progression or treatment limiting toxicity: clinical outcomes in advanced melanoma. Annals of Oncology (2019) 30:1154–1161. doi: 10.1093/annonc/mdz110.
17. Whitman ED, Scherrer E, Ou W, Krepler C. Outcomes of retreatment with anti-PD-1 monotherapy after response to first course in patients with cutaneous melanoma. Future Oncology (2020) 16:1441–1453. doi: 10.2217/fon-2020-0314.
18. Pires da Silva I, Ahmed T, Reijers ILM, Weppler AM, Betof Warner A, Patrinely JR, *et al.* Ipilimumab alone or ipilimumab plus anti-PD-1 therapy in patients with metastatic melanoma resistant to anti-PD-(L)1 monotherapy: a multicentre, retrospective, cohort study. Lancet Oncol (2021) 22:836–847. doi: 10.1016/S1470-2045(21)00097-8.
19. Asher N, Israeli-Weller N, Shapira-Frommer R, Ben-Betzalel G, Schachter J, Meirson T, *et al.* Immunotherapy Discontinuation in Metastatic Melanoma: Lessons from Real-Life Clinical Experience. Cancers (2021) 13:3074. doi: 10.3390/cancers13123074.
20. Pokorny R, McPherson JP, Haaland B, Grossmann KF, Luckett C, Voorhies BN, *et al.* Real-world experience with elective discontinuation of PD-1 inhibitors at 1 year in patients with metastatic melanoma. J Immunother Cancer (2021) 9:e001781. doi: 10.1136/jitc-2020-001781.
21. Persa OD, Mauch C. Outcomes after retreatment with MAPK inhibitors and immune checkpoint inhibitors in melanoma patients. Future Oncol (2021) 17:3809–3817. Outcomes after retreatment with MAPK inhibitors and immune checkpoint inhibitors in melanoma patients.
22. Hepner A, Atkinson VG, Larkin J, Burrell RA, Carlino MS, Johnson DB, *et al.* Re-induction ipilimumab following acquired resistance to combination ipilimumab and anti–PD-1 therapy. European Journal of Cancer (2021) 153:213–222. doi: 10.1016/j.ejca.2021.04.021.
23. van Zeijl MCT, van den Eertwegh AJM, Wouters MWJM, de Wreede LC, Aarts MJB, van den Berkmortel FWPJ, *et al.* Discontinuation of anti-PD-1 monotherapy in advanced melanoma-Outcomes of daily clinical practice. Int J Cancer (2022) 150:317–326. doi: 10.1002/ijc.33800.
24. Nardin C, Hennemann A, Diallo K, Funck-Brentano E, Puzenat E, Heidelberger V, *et al.* Efficacy of Immune Checkpoint Inhibitor (ICI) Rechallenge in Advanced Melanoma Patients’ Responders to a First Course of ICI: A Multicenter National Retrospective Study of the French Group of Skin Cancers (Groupe de Cancérologie Cutanée, GCC). Cancers (Basel) (2023) 15:3564. doi: 10.3390/cancers15143564.
25. Gobbini E, Toffart AC, Pérol M, Assié JB, Duruisseaux M, Coupez D, *et al.* Immune Checkpoint Inhibitors Rechallenge Efficacy in Non–Small-Cell Lung Cancer Patients. Clinical Lung Cancer (2020) 21:e497–e510. doi: 10.1016/j.cllc.2020.04.013.
26. Herbst RS, Garon EB, Kim DW, Cho BC, Perez-Gracia JL, Han JY, *et al.* Long-Term Outcomes and Retreatment Among Patients With Previously Treated, Programmed Death-Ligand 1‒Positive, Advanced Non‒Small-Cell Lung Cancer in the KEYNOTE-010 Study. J Clin Oncol (2020) 38:1580–1590. doi: 10.1200/JCO.19.02446.
27. Niki M, Nakaya A, Kurata T, Yoshioka H, Kaneda T, Kibata K, *et al.* Immune checkpoint inhibitor re-challenge in patients with advanced non-small cell lung cancer. Oncotarget (2018) 9:32298–32304. doi: 10.18632/oncotarget.25949.
28. Fujita K, Uchida N, Kanai O, *et al.* Retreatment with pembrolizumab in advanced non-small cell lung cancer patients previously treated with nivolumab: emerging reports of 12 cases. Cancer Chemother Pharmacol (2018) 81:1105-1109. doi: 10.1007/s00280-018-3585-9.
29. Fujita K, Uchida N, Kanai O, Okamura M, Nakatani K, Mio T*.* Retreatment With Anti-PD-L1 Antibody in Advanced Non-small Cell Lung Cancer Previously Treated With Anti-PD-1 Antibodies. Anticancer Res (2019) 39:3917-3921. doi: 10.21873/anticanres.13543.
30. Katayama Y, Shimamoto T, Yamada T, Takeda T, Yamada T, Shiotsu S, *et al.* Retrospective Efficacy Analysis of Immune Checkpoint Inhibitor Rechallenge in Patients with Non-Small Cell Lung Cancer. J Clin Med (2019) 9:102. doi: 10.3390/jcm9010102.
31. Watanabe H, Kubo T, Ninomiya K, Kudo K, Minami D, Murakami E, *et al.* The effect and safety of immune checkpoint inhibitor rechallenge in non-small cell lung cancer. Jpn J Clin Oncol (2019) 49:762–765. doi: 10.1093/jjco/hyz066.
32. Mouri A, Kaira K, Yamaguchi O, Shiono A, Miura Y, Hashimoto K, *et al.* Clinical difference between discontinuation and retreatment with nivolumab after immune-related adverse events in patients with lung cancer. Cancer Chemother Pharmacol (2019) 84:873–880. doi: 10.1007/s00280-019-03926-y.
33. Sternschuss M, Peled N, Allen AM, Dudnik E, Rotem O, Kurman N, *et al.* Can Ipilimumab restore immune response in advanced NSCLC after progression on anti-PD-1/PD-L1 agents? Thoracic Cancer (2020) 11:2331–2334. doi: 10.1111/1759-7714.13502.
34. Kitagawa S, Hakozaki T, Kitadai R, Hosomi Y*.* Switching administration of anti-PD-1 and anti-PD-L1 antibodies as immune checkpoint inhibitor rechallenge in individuals with advanced non-small cell lung cancer: Case series and literature review. Thorac Cancer (2020) 11:1927–1933. doi: 10.1111/1759-7714.13483.
35. Furuya N, Nishino M, Wakuda K, Ikeda S, Sato T, Ushio R, *et al.* Real-world efficacy of atezolizumab in non-small cell lung cancer: A multicenter cohort study focused on performance status and retreatment after failure of anti-PD-1 antibody. Thorac Cancer (2021) 12:613–618. doi: 10.1111/1759-7714.13824.
36. Takahara Y, Tanaka T, Ishige Y, Shionoya I, Yamamura K, Sakuma T, *et al.* Efficacy and predictors of rechallenge with immune checkpoint inhibitors in non-small cell lung cancer. Thoracic Cancer (2022) 13:624–630. doi: 10.1111/1759-7714.14309.
37. Yang J, Zeng R, Zhou J, Luo L, Lyu M, Liu F, *et al.* Efficacy, prognosis and safety analysis of anti-PD-1/PD-L1 inhibitor rechallenge in advanced lung cancer patients: a cohort study. Transl Lung Cancer Res (2022) 11:1038–1050. doi: 10.21037/tlcr-22-360.
38. Akamatsu H, Teraoka S, Takamori S, Miura S, Hayashi H, Hata A, *et al.* Nivolumab Retreatment in Non-Small Cell Lung Cancer Patients Who Responded to Prior Immune Checkpoint Inhibitors and Had ICI-Free Intervals (WJOG9616L). Clin Cancer Res (2022) 28:OF1–OF7. doi: 10.1158/1078-0432.CCR-22-0602.
39. Bilger G, Girard N, Doubre H, Levra MG, Giroux-Leprieur E, Giraud F, *et al.* Discontinuation of immune checkpoint inhibitor (ICI) above 18 months of treatment in real-life patients with advanced non-small cell lung cancer (NSCLC): INTEPI, a multicentric retrospective study. Cancer Immunol Immunother (2022) 71:1719–1731. doi: 10.1007/s00262-021-03114-z.
40. Xu Z, Hao X, Yang K, Wang Q, Wang J, Lin L, *et al.* Immune checkpoint inhibitor rechallenge in advanced or metastatic non-small cell lung cancer: a retrospective cohort study. J Cancer Res Clin Oncol (2022) 148:3081–3089. doi: 10.1007/s00432-021-03901-2.
41. Torasawa M, Yoshida T, Takeyasu Y, Shimoda Y, Tateishi A, Matsumoto Y, *et al.* Disease progression status during initial immune checkpoint inhibitor (ICI) affects the clinical outcome of ICI retreatment in advanced non-small cell lung cancer patients. Cancer Med (2023) 12:12388–12401. doi: 10.1002/cam4.5939.
42. Gul A, Stewart TF, Mantia CM, Shah NJ, Gatof ES, Long Y, *et al.* Salvage Ipilimumab and Nivolumab in Patients With Metastatic Renal Cell Carcinoma After Prior Immune Checkpoint Inhibitors. J Clin Oncol (2020) 38:3088–3094. doi: 10.1200/JCO.19.03315.
43. Abou Alaiwi S, Xie W, Nassar AH, Dudani S, Martini D, Bakouny Z, *et al.* Safety and efficacy of restarting immune checkpoint inhibitors after clinically significant immune-related adverse events in metastatic renal cell carcinoma. J Immunother Cancer (2020) 8:e000144. doi: 10.1136/jitc-2019-000144.
44. Ravi P, Mantia C, Su C, Sorenson K, Elhag D, Rathi N, *et al.* Evaluation of the Safety and Efficacy of Immunotherapy Rechallenge in Patients With Renal Cell Carcinoma. JAMA Oncol (2020) 6:1606–1610. doi: 10.1001/jamaoncol.2020.2169.
45. Lee CH, Shah AY, Rasco D, Rao A, Taylor MH, Di Simone C, *et al.* Lenvatinib plus pembrolizumab in patients with either treatment-naive or previously treated metastatic renal cell carcinoma (Study 111/KEYNOTE-146): a phase 1b/2 study. Lancet Oncol (2021) 22:946–958. doi: 10.1016/S1470-2045(21)00241-2.
46. Choueiri TK, Kluger H, George S, Tykodi SS, Kuzel TM, Perets R, *et al*. FRACTION-RCC: nivolumab plus ipilimumab for advanced renal cell carcinoma after progression on immuno-oncology therapy. J Immunother Cancer (2022) 10:e005780. doi: 10.1136/jitc-2022-005780.
47. Vauchier C, Auclin E, Barthélémy P, Carril-Ajuria L, Ryckewaert T, Borchiellini D, *et al.* REchallenge of NIVOlumab (RENIVO) or Nivolumab-Ipilimumab in Metastatic Renal Cell Carcinoma: An Ambispective Multicenter Study. J Oncol (2022) 2022:3449660. doi: 10.1155/2022/3449660.
48. Pal SK, Albiges L, Tomczak P, Suárez C, Voss MH, de Velasco G, *et al.* Atezolizumab plus cabozantinib versus cabozantinib monotherapy for patients with renal cell carcinoma after progression with previous immune checkpoint inhibitor treatment (CONTACT-03): a multicentre, randomised, open-label, phase 3 trial. The Lancet (2023) 402:185–195. doi: 10.1016/S0140-6736(23)00922-4.
49. Bernard-Tessier A, Baldini C, Martin P, Champiat S, Hollebecque A, Postel-Vinay S, *et al.* Outcomes of long-term responders to anti-programmed death 1 and anti-programmed death ligand 1 when being rechallenged with the same anti-programmed death 1 and anti-programmed death ligand 1 at progression. European Journal of Cancer (2018) 101:160–164. doi: 10.1016/j.ejca.2018.06.005.
50. Simonaggio A, Michot JM, Voisin AL, Le Pavec J, Collins M, Lallart A, *et al.* Evaluation of Readministration of Immune Checkpoint Inhibitors After Immune-Related Adverse Events in Patients With Cancer. JAMA Oncol (2019) 5:1310–1317. doi: 10.1001/jamaoncol.2019.1022.
51. Sheth S, Gao C, Mueller N, Angra N, Gupta A, Germa C, *et al*. Durvalumab activity in previously treated patients who stopped durvalumab without disease progression. J Immunother Cancer (2020) 8:e000650. doi: 10.1136/jitc-2020-000650.
52. Wong JSL, Kwok GGW, Tang V, Li BCW, Leung R, Chiu J, *et al.* Ipilimumab and nivolumab/pembrolizumab in advanced hepatocellular carcinoma refractory to prior immune checkpoint inhibitors. J Immunother Cancer (2021) 9:e001945. doi: 10.1136/jitc-2020-001945.
53. Scheiner B, Roessler D, Phen S, Lim M, Pomej K, Pressiani T, *et al.* Efficacy and safety of immune checkpoint inhibitor rechallenge in individuals with hepatocellular carcinoma. JHEP Rep (2022) 5:100620. doi: 10.1016/j.jhepr.2022.100620.
54. Ito K, Kita Y, Yokomizo A, Miki J, Yoshio Y, Matsumoto H, *et al.* Discontinuation of pembrolizumab for advanced urothelial carcinoma without disease progression: Nationwide cohort study. Cancer Med (2022) 12:2325–2332. doi: 10.1002/cam4.5057.
55. Makrakis D, Bakaloudi DR, Talukder R, Lin GI, Diamantopoulos LN, Jindal T, *et al.* Treatment Rechallenge With Immune Checkpoint Inhibitors in Advanced Urothelial Carcinoma. Clin Genitourin Cancer (2023) 21:286–294. doi: 10.1016/j.clgc.2022.11.003.
56. Zhang XX, Yang XF, Li S, Wu C, Hou XF*.* Clinical analysis of immunotherapy rechallenge in advanced gastric cancer. Zhonghua Zhong Liu Za Zhi (2023) 45:605-612. doi: 10.3760/cma.j.cn112152-20220418-00261.
57. Li G, Cheng M, Hong K, Jiang Y*.* Clinical Efficacy and Safety of Immunotherapy Retreatment in Metastatic Cervical Cancer: A Retrospective Study. OTT Volume (2023) 16:157–163. doi: 10.2147/OTT.S400376.
58. Wakasugi T, Takeuchi S, Ohkubo JI, Suzuki H*.* Retreatment with nivolumab for patients with recurrent and/or metastatic head and neck cancer. Acta Otolaryngol (2022) 142:206–212. doi: 10.1080/00016489.2022.2033317.
59. Ding X, Hua YJ, Zou X, Chen XZ, Zhang XM, Xu B, *et al*. Camrelizumab plus famitinib in patients with recurrent or metastatic nasopharyngeal carcinoma treated with PD-1 blockade: data from a multicohort phase 2 study. ClinicalMedicine (2023) 61:102043. doi: 10.1016/j.eclinm.2023.102043.
60. Weppler AM, Da Meda L, Pires da Silva I, Xu W, Grignani G, Menzies AM, *et al.* Durability of response to immune checkpoint inhibitors in metastatic Merkel cell carcinoma after treatment cessation. Eur J Cancer (2023) 183:109–118. doi: 10.1016/j.ejca.2023.01.016.
